# Supplementary material for: Electronic cigarette use (vaping) and patterns of tobacco cigarette smoking in pregnancy–evidence from a population-based maternity survey in England
Source: PLoS One. 2021 Jun 4;16(6):e0252817. doi: 10.1371/journal.pone.0252817 (PMC8177470; doi:10.1371/journal.pone.0252817)
Supplement: Attachment — Submitted filename: PLOS One review.3.3.21.docx [file pone.0252817.s001.docx]

**PONE-D-21-04030**

This paper is an interesting retrospective analysis of the prevalence of e-cigarette use during pregnancy among UK women who gave birth between the 15^th^ and 28^th^ of October 2017. The large nationally representative sample strengthens the study, and the groups identified via the LCA are nicely labeled (however the methods for how the LCA was conducted could improve). However, there are typos and minor errors throughout the paper that do need to be addressed, and the introduction and discussion need major revisions to align better with the focus of the paper. Overall, this seems to be an important study that will add valuable information to literature, but I hope the authors will consider the following recommendations for revision.

**Abstract:**

Please include the N value in the abstract.

Should it read “…and regression analysis was used to explore….”?

The conclusions currently read as though they are an extension of the results. What are the major take-aways that can provide to those working with pregnant women who smoke and vape or who smoke and are considering vaping?

**Introduction:**

Some of the introduction is a bit choppy. Also, some areas are vague. For example, it says, “The prevalence of smoking in pregnancy has also declined slightly in some places while remaining

relatively stable in others.” It would be helpful to provide (e.g., …) to help the reader know where ‘some places’ are.

Also, the introduction goes a bit too deep into the overall population who vapes. It would be more helpful to focus the paper only on vaping during pregnancy. There are several new articles the authors have not included that help explain why women might vape during pregnancy and transitions from smoking to vaping during pregnancy (Kurti et al, 2020). Currently, the paragraph about the transitioning seems to be about young adults’ transitions from vaping to cigarette use. While I do agree the literature shows this occurs, I do not think that literature is about pregnant women. However, there are articles about pregnant smokers who using e-cigarettes.

Although I agree with the authors on the way they have framed the paper and do avoid suggesting that e-cigarettes are a safer alternative to smoking for pregnant women (due to nicotine exposure that can be harmful to the fetus); however, the paper is missing at least the discussion of harm reduction. There appears to be at least some literature about pregnant women using e-cigarettes as a harm reduction method. The introduction could use a little of this to provide balance or at least some understanding of what may influence this behavior among pregnant women who smoke. Also, there appear to be some articles that discuss prevalence that are not cited. While the current study would likely provide one of the best samples to demonstrate prevalence of e-cigarette use during pregnancy in the UK, the introduction needs to make a case for the need for this study. Moreover, nothing in the introduction includes breastfeeding and smoking and/or vaping during pregnancy. It is not until the discussion that this is mentioned. This should at least be mentioned in the introduction to help understand why this variable was included.

Some suggested articles include:

Kurti et al. (2020). Impact of electronic nicotine delivery systems and other respondent

characteristics on tobacco use transitions among a U.S. national sample of women of

reproductive age. *Drug and Alcohol Dependence*, 207, 107801

McCubbin et al. (2020). Perceptions, Characteristics, and Behaviors of Cigarette and

Electronic Cigarette Use among Pregnant Smokers. *Women’s Health Issues,* 30-3, 221-

229.

Froggatt et al. (2021). Risk perception of cigarette and e-cigarette use during pregnancy: A

qualitative postpartum perspective. *Midwifery*, 94, 102917

Wagner et al. (2017). Prevalence and Perceptions of Electronic Cigarette Use during Pregnancy.

*Maternal and Child Health Journal*, 21, 1655-1661.

Dobbs et al. (2020). Gestational Women’s Perceptions About the Harms of Cigarette and

E‑Cigarette Use During Pregnancy. *Maternal and Child Health Journal.*

doi.org/10.1007/s10995-020-03085-0

Cooper et al. (2017). Smoking and quit attempts during pregnancy and postpartum: a

longitudinal UK cohort. *BMJ Open*, 7, e018746.

Schilling et al. (2020). STudy on E-Cigarettes and Pregnancy (STEP) – Study Protocol of a

Mixed Methods Study on Risk Perception of E-Cigarette Use During Pregnancy and

Sample Description. *Geburtshilfe Frauenheilkd*, 80(1), 66-75.

Schilling et al. (2019). Perceived threats, benefits and barriers of e-cigarette use during

pregnancy. A qualitative analysis of risk perception within existing threads in online

discussion forums. *Midwifery*, 79, 102533.

Currently, the introduction needs major revisions to frame the need for the study with the context of the target population. Other than two disconnected sentences about tobacco use and smoking during pregnancy in the first paragraph, I might assume the paper was going to be about young adult e-cigarette use until the final paragraph of the introduction that briefly mentions vaping during pregnancy without citations. Please consider rewriting the introduction to focus on the need for information about the prevalence of vaping during pregnancy and the need for more information about health outcomes that are associated with vaping during pregnancy.

**Methods:**

Once I read the findings from the LCA in the results, the groups made intuitive sense; however, reading about the LCA in the analysis was confusing. I wasn’t sure which items were used or how the classes were identified. It was confusing when it mentions the five latent classes from a previous study. If the classes were already identified, why do an LCA? Also, what model-fit indices were used, and how did the authors decide upon the identified classes? Why was SEM used after the LCA?

It would be interesting if the authors explored associations between maternal smoking and vaping (separately) and birth outcomes and breastfeeding. The sample size of those who only vaped during pregnancy may be too small, but analyzing those who smoked but did not vape and their outcomes and breastfeeding may provide a comparison to other studies that could strengthen the validity of the findings. Currently, it seems as though maternal vaping and dual vaping/smoking does not seem to be associated with any immediate concerns in regard to birth outcomes and breastfeeding.

Instead of binomial regression, please use logistic regression.

Patient and public involvement seems out of place. This likely needs to be moved up to explain the study procedures.

**Results:**

Instead of age when left full-time education, do you have data for education completed?

The term ‘deprived areas’ is first mentioned in the results. What was considered a ‘deprived area’? This should be explained in the methods, measures section.

The percentage of women who smoked between the three months prior to pregnancy and six months postpartum seems high. For this reason, conducting the outcomes analysis with only smokers (who did not report vaping) would be interesting.

**Discussion:**

While the discussion is interesting, it seems to repeat the results in the first two sections. Confident intervals are unnecessary in the discussion.

While it is important to include what the paper adds to literature, we need to know a little about what currently exists to know what gaps in literature this paper fills. The discussion doesn’t begin to compare findings to others’ research until the third paragraph. Return to the second paragraph to see what is necessary, and consider removing the rest. It would help the reader stay engaged if you get to the point. Point out differences between your findings and others and then highlight what gap in literature was closed by your findings. Using literature, then explain why this is important.

It is interesting that your findings suggest no concerns about negative health outcomes associated with vaping during pregnancy; however, the authors recommend “the development of alternative smoking cessation or reduction tools for particular use in pregnancy which limit harms to mother and baby.” First, what is a reduction tool? I wasn’t sure if the authors meant a harm reduction tool at first, but then thought this was referring to cessation methods. Next, if the authors found no associations with vaping during pregnancy and harm, why still suggest not using these devices? Unless the authors show the association between smoking during pregnancy and adverse health outcomes, it is unclear why the co-use of smoking and vaping could increase the prevalence of adverse pregnancy and birth health outcomes.

**Minor comments:**
Where it reads ‘raises a real risk…’ please remove ‘real’.

Some areas are wordy and confusing. For example, where it reads ‘and with that the emergence of reports of’, the authors could simply use ‘followed by reports of…’

Commas appeared to be misused throughout the paper. For example, “…during pregnancy, and to women planning a pregnancy.” Also, the purpose statement does not need a comma. To use a common after the word “and”, there must be a noun and verb on both sides of the comma.

The last sentence before analysis is missing punctuation.

What does ‘explained away’ mean?

Avoid using terms such as ‘very’.

Again, some wordy sections included “It may also indicate women who were more frequent smokers were turning to vaping to aid smoking cessation but not being successful at quitting.” This could be rephrased to read “This may indicate participants who were more frequent smokers attempted to use vaping to quit smoking but were unsuccessful.”
