# Supplementary material for: Electronic cigarette use (vaping) and patterns of tobacco cigarette smoking in pregnancy–evidence from a population-based maternity survey in England
Source: PLoS One. 2021 Jun 4;16(6):e0252817. doi: 10.1371/journal.pone.0252817 (PMC8177470; doi:10.1371/journal.pone.0252817)
Supplement: Attachment — Submitted filename: Response to reviewers comments.docx [file pone.0252817.s002.docx]

| **Comments** | **Response** |
| --- | --- |
| **Editors** |  |
| Please ensure that your manuscript meets PLOS ONE's style requirements, including those for file naming. The PLOS ONE style templates can be found at: <https://journals.plos.org/plosone/s/file?id=wjVg/PLOSOne_formatting_sample_main_body.pdf> and  <https://journals.plos.org/plosone/s/file?id=ba62/PLOSOne_formatting_sample_title_authors_affiliations.pdf> |  |
| Please provide a sample size and power calculation in the Methods, or discuss the reasons for not performing one before study initiation. | We have added a ‘sample size’ section to the methods. In the process we have added a citation for the study from which some information relating to the calculation was obtained and removed ’16,000’ from the ‘data sources and acquisition’ section. |
| Please state whether the questionnaire provided participants with information about the study and how their responses would be used. Please also state whether participants were required to provide written informed consent to be included in the study. | A participant information leaflet was enclosed with the questionnaire, which provided detailed information about the study and how the responses from participants would be used. Completion and return/submission of the postal/online questionnaire was taken as implicit consent to participate in the study. We have added this clarification in the methods and further indicated that this method was specifically reviewed and approved by the ethics committee. (The following statement regarding consent was submitted in the ethics application: *‘As with all of the previous National Maternity Surveys, questionnaire return will be taken as implicit consent. The participant information sheet will make it clear that participation is entirely voluntary and that women will be free to withdraw their participation from the study by contacting the research team if they change their minds after completion and return of the questionnaire.’*) |
| In the ethics statement in the manuscript Methods, please explain why ethics approval was obtained from a review board that is not associated with your institution. | We were advised by our Clinical Trials and Research Governance Team to obtain ethics approval through an NHS Research Ethics Committee (REC) as opposed to through Oxford Central University Research Ethics Committee (CUREC). The specific review board was selected because it was the next available meeting. We have added this clarification under ‘ethical approval’. |
| We note that you have indicated that data from this study are available upon request. PLOS only allows data to be available upon request if there are legal or ethical restrictions on sharing data publicly. For information on unacceptable data access restrictions, please see <http://journals.plos.org/plosone/s/data-availability#loc-unacceptable-data-access-restrictions>. If there are ethical or legal restrictions on sharing a de-identified data set, please explain them in detail (e.g., data contain potentially identifying or sensitive patient information) and who has imposed them (e.g., an ethics committee). Please also provide contact information for a data access committee, ethics committee, or other institutional body to which data requests may be sent. If there are no restrictions, please upload the minimal anonymized data set necessary to replicate your study findings as either Supporting Information files or to a stable, public repository and provide us with the relevant URLs, DOIs, or accession numbers. Please see http://www.bmj.com/content/340/bmj.c181.long for guidelines on how to de-identify and prepare clinical data for publication. For a list of acceptable repositories, please see <http://journals.plos.org/plosone/s/data-availability#loc-recommended-repositories>. We will update your Data Availability statement on your behalf to reflect the information you provide. | We have edited the ‘data availability’ section to explain that restrictions on sharing are imposed by the fact that the ONS is the controller of part of the data therefore we cannot legally share it, and limits on the scope of consent obtained from participants. We have also provided an email contact address for the NPEU. |
| Your ethics statement should only appear in the Methods section of your manuscript. If your ethics statement is written in any section besides the Methods, please move it to the Methods section and delete it from any other section. Please ensure that your ethics statement is included in your manuscript, as the ethics statement entered into the online submission form will not be published alongside your manuscript. | We have moved the ‘ethical approval’ section to the methods and deleted it elsewhere. |
|  |  |
| **Reviewer 1** |  |
| This study involved a cross-sectional population-based postal survey (N=4421) of maternal and infant health and examined the prevalence of vaping and associations between vaping and maternal characteristics. Vaping has the potential for both maternal and infant harm as well as for aiding smoking cessation; therefore, it is important to know about both the prevalence of vaping and factors associated with vaping. This is a rigorously conducted study and is likely to have a more representative sample than the previous similar survey by Bowker and colleagues (ref 33). The conclusions seem well justified and I consider this a valuable contribution to the literature. I only have a couple of suggestions relating to the referencing of related literature. | Thank you for this comment. |
| I think far too much of the introduction is spent discussing smoking and vaping in the general population and the authors need to place the study more in the context of the growing literature on vaping in pregnancy. This very recent review might be helpful: Robert Calder, PhD, Eleanor Gant, MSc, Linda Bauld, PhD, Ann McNeill, PhD, Debbie Robson, PhD, Leonie S Brose, PhD, Vaping in pregnancy: A systematic review Nicotine & Tobacco Research https://doi.org/10.1093/ntr/ntab017 Published: 04 February 2021 | We have now included evidence from this review in the introduction and highlighted the limited evidence on the topic in a paragraph of the introduction. |
| When the authors mention “a systematic review exploring smoking cessation among electronic cigarette users found lower odds of success of quitting cigarettes among vapers, whether or not they took up vaping with an intention to quit smoking36.”, I suggest they offer a more balanced perspective by also referring to the findings of the recent Cochrane review on vaping for smoking cessation: https://www.cochranelibrary.com/cdsr/doi/10.1002/14651858.CD010216.pub4/full | We have included the findings of the suggested review in the discussion to offer a balanced perspective. |
|  |  |
| **Reviewer 2** |  |
| The findings from the LCA are not reported, other than saying classes were formed. What model fit indices were used to determine the classes, and where were the findings from the LCA reported? Also, the paper includes several typographical errors and needs revisions throughout (abstract to discussion) to address these. | We have clarified in the description of the LCA on page 6 of 23 that this was a confirmatory model in which, rather than comparing the fit of alternative models based on their fit indices, we applied constraints based on previous empirical evidence (which we have cited in the description) to group women in five latent classes. The key findings of the LCA are reported in Figure 2. We have corrected the various typographical errors identified in the manuscript. |
|  |  |
| **Reviewer 3** |  |
| This paper examined the prevalence of e-cigarette use (vaping) during pregnancy among a cohort of women in England and the associations between vaping and birth outcomes and breastfeeding. This is an important area as there is limited known about vaping in this population as well as the impact on infant health. While the authors have produced a compelling paper, I would like to raise two methodological points. | Thank you for this comment. |
| First, I would like to comment upon how the authors have structured adjusting in their models in Tables 2-4. There is a substantial body of research establishing the associations between smoking cigarettes with socio-demographic characteristics and birth outcomes. Rather than adjusting for smoking patterns in the ‘further adjusted model’, I would suggest they consider only adjusting for cigarette use before they adjust for the additional socio-demographic characteristics. This would then isolate the effect of vaping independent of smoking on these characteristics. It is not currently clear why adjusting for smoking is left to the ‘further adjusted model’ as smoking is likely the main driver for many of these associations. | We have now added a new column to Tables 3 and 4 in which we present effects adjusted for smoking patterns only before the further adjusting for sociodemographic characteristics. Table 2 includes a row for the univariate effect of smoking and adjusted effects in the ‘further adjusted’ column. |
| Second, the authors commented on page 7 that because the variables country of birth and ethnicity were ‘correlated’ then they have chosen to only include country of birth in their model. It would helpful if the authors produced a table or describe in text how much overlap there is between the two variables. As smoking is known to vary by ethnicity, it seems the authors are missing the opportunity to look at this important risk factor. One suggestion is to run sensitivity analyses and substitute ‘ethnicity’ for ‘country of birth’ to see whether and how the associations change between these two approaches. | We have added in the text the polychoric correlation coefficient for between country of birth and ethnicity and p-value for this correlation. It shows a very high degree of correlation which suggests that adjusting for one or the other is sufficient to capture the role of this construct in the association (we are not specifically interested in the effect of either variable). We have explained in the text why we have subsequently chosen to adjust for country of birth, including more missingness in ethnicity data and very few observations in some groups which would result in exclusion of nearly 10% more observations in sensitivity analyses, making it difficult to compare these results with the main analyses. |
| Abstract • The authors should include the N of the survey • The results report that there was no association between vaping and breastfeeding; however, the conclusion states that ‘reduced breastfeeding among women…’ While this is more clearly described in the results (in the paper), this sounds contradictory in the abstract | We have added the number of women who responded to the survey to the first line of results in the abstract.  We have removed the statement about breastfeeding from the conclusions in the abstract. |
| Introduction • While the authors introduce 1 study on the prevalence of e-cigarette use during pregnancy in the US in the discussion, I think the authors should include a paragraph describing the limited research in this area related to the prevalence and associations with birth outcomes. The authors should also consider citing Calder et al. Nicotine & Tobacco Research 2021: Vaping in pregnancy: A systematic review | We have now cited the Calder review in the introduction, highlighting the limited research on this topic. |
| Methods • A limitation of the collection of vaping and smoking data is how the questions were asked. In the limitations section (in discussion), the authors should note that there is no information on the quantity of consumption (i.e. how many times vaped during that time period), type of device used, and level of nicotine contained in the vaping products. Also, was the number of cigarettes smoked per day collected? From this description it does not appear so, but this would also be a limitation that should be acknowledged. | We have added to the limitations an acknowledgement that information on frequency, quantity, type of vaping device or level of nicotine in vaping products was not collected.  We have also included in the limitations that the number of cigarettes smoked per day was not collected. |
| • The socio-demographic characteristics listed in the paragraph do not match all that were included in Table 1, including education and index of multiple deprivation. The last sentence comments that the birth outcomes and breastfeeding were collected in the survey. From this statement it is not clear if this information was also collected by self-report. If this is the case, then that should be noted as birth weight and gestational age are not likely accurately recalled and should be noted in the limitations section. | We have added IMD and education to this paragraph.  We have also added the word ‘questionnaire’ to the end of the paragraph describing variables to be clear that all this information was collected by self-report in the survey. We have indicated in the limitations that self-reported outcomes could be subject to recall bias. |
| • The authors should include the definition of preterm birth. | We have included a definition of preterm birth in the variables section. It is defined as a birth occurring before 37 complete weeks of gestation. |
| • It would also be helpful to know whether the birth outcome data were cleaned, including excluding implausible birth weights for gestational age or any other exclusions of the data such as age restrictions or how missing data were handled. | We have now described in the methods at the end of the variables section how this was done. No other data were excluded. The amount of missing data was low (<3%) across all variables used in the current analysis and all available data for other variables were used. |
| • It would be helpful if the authors could clarify why they chose binomial regression models rather than logistic regression models for the dichotomous outcomes. | Binomial regression models were selected because we chose to report magnitudes of association as risk ratios. Logistic regression models estimate odds ratios and require an extra step to convert these to risk ratios, which are identical to those obtained from binomial regression models. |
| • To clarify, the authors mention that weights were included to adjust for non-response and thus make the results nationally-representative, correct? Or simply to adjust for non-response. | Yes, both of these statements are correct – the sampling frame was all births nationally at a point in time, and therefore adjusting for non-response makes the data more representative of the population. Weights were used to make each observation’s contribution to the overall estimates proportional to the degree to which their set of characteristics was underrepresented or overrepresented in the sample, to result in overall estimates more representative of the distribution of individual characteristics in the national population. We have added this clarification in the analysis section. |
| Results • The first paragraph states ‘married’ but the table indicates the definition was ‘living with partner’ | This was in error – we have replaced ‘married’ with ‘living with a partner’ in the paragraph. |
| • On page 8, the authors note that the pattern of smoking could not be determined in 24 women but Table 1 still includes these women. It seems they should be excluded from the analytic sample as they will not be included in the adjusted regression models. | Women for whom smoking patterns could not be determined are only excluded from analyses adjusting for smoking pattern. It is often the case that adjusted models may include fewer women than unadjusted models due to missingness in covariates, and it is not standard practice to base the descriptive table on only the set of women included in the adjusted model (i.e. those with complete data on all covariates). |
| • On page 9, it is not clear why ‘ex-smokers’ is the baseline group for the smoking pattern variable (Table 2). Shouldn’t everyone be compared to ‘never-smokers’? | Comparing everyone to ‘never-smokers’ is one option for choice of reference group, as is comparing to the highest level, ‘persistent smokers’. Both choices are based on a strategy of comparison to a normative group. There are other strategies for choosing a reference group, including the use of the largest group, or the use of a group with a mean or frequency near the middle of the spread of means or frequencies across the groups. We have used this latter strategy to avoid nominally extreme relative risks. None of these strategies affects the evidence for or the magnitudes or directions of association. |
| Discussion • On page 13, the authors only describe 1 study from the US that examined the prevalence of prenatal e-cigarette use and there are multiple studies published on this topic. While a comprehensive review is not needed, it would be helpful to describe existing studies more fully. | We have now discussed evidence from four other studies on this topic including a new review which cites some of the studies we have already cited. |
| • The discussion describes the association between vaping and smoking and I think adjusting for smoking first would contribute to the points raised, particularly in relation to the birth outcomes and breastfeeding. | We have now added a column to Tables 3 and 4 showing the estimates adjusted for smoking pattern only, before other factors. |
| • As noted previously, the limitations section should be expanded to comment upon how the birth outcomes were collected and the information that wasn’t collected in the questions related to vaping and smoking noted previously. | This has now been added to the limitations.  We have also edited the discussion to mention limitations relating to how the birth outcomes were collected and the information relating to vaping and smoking that was not collected. |
|  |  |
| **Reviewer 4** |  |
| This paper is an interesting retrospective analysis of the prevalence of e-cigarette use during pregnancy among UK women who gave birth between the 15^th^ and 28^th^ of October 2017. The large nationally representative sample strengthens the study, and the groups identified via the LCA are nicely labeled (however the methods for how the LCA was conducted could improve). However, there are typos and minor errors throughout the paper that do need to be addressed, and the introduction and discussion need major revisions to align better with the focus of the paper. Overall, this seems to be an important study that will add valuable information to literature, but I hope the authors will consider the following recommendations for revision. | Thank you for this comment. The specific issues you raise (e.g. more details on the LCA, typos, minor errors, introduction, discussion) are responded to below and in response to other reviewers’ comments. |
| **Abstract:**  Please include the N value in the abstract. | This has now been added in the results section of the abstract. |
| Should it read “…and regression analysis was used to explore….”? | We have re-worded this sentence in the ‘methods’ section of the abstract as suggested. |
| The conclusions currently read as though they are an extension of the results. What are the major take-aways that can provide to those working with pregnant women who smoke and vape or who smoke and are considering vaping? | We have now removed the last line of the conclusions and replaced it with a statement about the co-occurrence of vaping and smoking and potential harm for pregnant women. |
| **Introduction:**  Some of the introduction is a bit choppy. Also, some areas are vague. For example, it says, “The prevalence of smoking in pregnancy has also declined slightly in some places while remaining  relatively stable in others.” It would be helpful to provide (e.g., …) to help the reader know where ‘some places’ are. | We have removed this sentence from the first paragraph of the introduction. We now introduce background information on vaping in pregnancy later in this section after laying out some historical and contextual information on vaping more generally and its specific relevance in pregnancy. |
| Also, the introduction goes a bit too deep into the overall population who vapes. It would be more helpful to focus the paper only on vaping during pregnancy. There are several new articles the authors have not included that help explain why women might vape during pregnancy and transitions from smoking to vaping during pregnancy (Kurti et al, 2020). Currently, the paragraph about the transitioning seems to be about young adults’ transitions from vaping to cigarette use. While I do agree the literature shows this occurs, I do not think that literature is about pregnant women. However, there are articles about pregnant smokers who using e-cigarettes. | We have removed some of the information about vaping in the general population and among young adults (e.g. at the end of the first paragraph and from most of the third paragraph). We have retained some of the descriptions of historical information about smoking and vaping to highlight part of the reason for the limited information on the topic. We have also cited the suggested study, although we feel that it is best suited to be included in the discussion rather than the introduction. |
| Although I agree with the authors on the way they have framed the paper and do avoid suggesting that e-cigarettes are a safer alternative to smoking for pregnant women (due to nicotine exposure that can be harmful to the fetus); however, the paper is missing at least the discussion of harm reduction. There appears to be at least some literature about pregnant women using e-cigarettes as a harm reduction method. The introduction could use a little of this to provide balance or at least some understanding of what may influence this behavior among pregnant women who smoke. Also, there appear to be some articles that discuss prevalence that are not cited. While the current study would likely provide one of the best samples to demonstrate prevalence of e-cigarette use during pregnancy in the UK, the introduction needs to make a case for the need for this study. Moreover, nothing in the introduction includes breastfeeding and smoking and/or vaping during pregnancy. It is not until the discussion that this is mentioned. This should at least be mentioned in the introduction to help understand why this variable was included. | We have introduced the concept of vaping for harm reduction in the third paragraph and cited more evidence around harm reduction in the discussion where the evidence around it was previously explored. While responding to other reviewers, we have also cited additional articles discussing prevalence of vaping. In the introduction, we have highlighted the limited geographical scope and size of currently published studies which justifies the need for this study, and also mentioned outcomes previously explored, including breastfeeding, birthweight, gestation etc. |
| Currently, the introduction needs major revisions to frame the need for the study with the context of the target population. Other than two disconnected sentences about tobacco use and smoking during pregnancy in the first paragraph, I might assume the paper was going to be about young adult e-cigarette use until the final paragraph of the introduction that briefly mentions vaping during pregnancy without citations. Please consider rewriting the introduction to focus on the need for information about the prevalence of vaping during pregnancy and the need for more information about health outcomes that are associated with vaping during pregnancy. | We have revised the introduction to reframe it more tightly around vaping and pregnancy. We have however retained some of the descriptions of historical information about smoking and vaping to highlight part of the reason for the limited information on the topic. |
| **Methods:**  Once I read the findings from the LCA in the results, the groups made intuitive sense; however, reading about the LCA in the analysis was confusing. I wasn’t sure which items were used or how the classes were identified. It was confusing when it mentions the five latent classes from a previous study. If the classes were already identified, why do an LCA? Also, what model-fit indices were used, and how did the authors decide upon the identified classes? Why was SEM used after the LCA? | We have clarified in the description of the LCA. The items used were women’s responses to the question about smoking frequency. The classes were identified using a confirmatory LCA model in which, rather than one comparing the fit of alternative models based on their fit indices, we applied the constraints according to previous empirical evidence cited in the description which suggested five latent classes. Thus, reporting the fit indices for this one model would not be informative as these indices are intended for comparing alternative models to identify the best fitting one – which was not our objective in this part of the analysis. The purpose of the LCA was to group women according to their smoking frequency. This section also explains that the SEM was used to conduct the LCA (not *after* the LCA), and that the role of the previous study was to inform on the number and identity of classes to explore in the LCA. |
| It would be interesting if the authors explored associations between maternal smoking and vaping (separately) and birth outcomes and breastfeeding. The sample size of those who only vaped during pregnancy may be too small, but analyzing those who smoked but did not vape and their outcomes and breastfeeding may provide a comparison to other studies that could strengthen the validity of the findings. Currently, it seems as though maternal vaping and dual vaping/smoking does not seem to be associated with any immediate concerns in regard to birth outcomes and breastfeeding. | We appreciate this suggestion; however, we note that our study is too small to separate out the effects of smoking and vaping as both were relatively rare exposures. We have not assessed the role of smoking on outcomes because it is a different research question from what this study intended to address. Many other studies have already explored this question and the evidence for the role of smoking on maternal and birth outcomes is discussed in the introduction. |
| Instead of binomial regression, please use logistic regression. | For the purpose of estimating risks and risk ratios, binomial regression models are identical to logistic regression but have the advantage that risks and risk ratios can be obtained in a single step, rather than a two-step procedure required with logistic regression which outputs odds and odds ratios which then need to be converted to risks and risk ratios. |
| Patient and public involvement seems out of place. This likely needs to be moved up to explain the study procedures. | We have reported patient and public involvement (PPI) details at the end of the methods section consistent with the reporting of other studies with PPI, for example, <https://bmjopen.bmj.com/content/10/5/e031589> and <https://bmjopen.bmj.com/content/9/7/e028227> |
| **Results:**  Instead of age when left full-time education, do you have data for education completed? | The questionnaire asked women about their age at the time when they left full-time education. We have used this variable to assess the level of education of respondents. |
| The term ‘deprived areas’ is first mentioned in the results. What was considered a ‘deprived area’? This should be explained in the methods, measures section. | We have now added in the methods that one of the participant characteristics is the index of multiple deprivation, a measure of area deprivation. We have also provided a reference for this. |
| The percentage of women who smoked between the three months prior to pregnancy and six months postpartum seems high. For this reason, conducting the outcomes analysis with only smokers (who did not report vaping) would be interesting. | We agree that this would be an interesting analysis; however, it is a different question from the focus of this research, which is vaping in pregnancy and its association with outcomes. The role of smoking on outcomes in pregnancy is historically well-described in other studies and we have already summarised some of the evidence in the introduction. |
| **Discussion:**  While the discussion is interesting, it seems to repeat the results in the first two sections. Confident intervals are unnecessary in the discussion. | In reporting this study, we have followed the STROBE guidelines which recommend that the key results be summarised (with reference to the study objectives) as the first part of the discussion. |
| While it is important to include what the paper adds to literature, we need to know a little about what currently exists to know what gaps in literature this paper fills. The discussion doesn’t begin to compare findings to others’ research until the third paragraph. Return to the second paragraph to see what is necessary, and consider removing the rest. It would help the reader stay engaged if you get to the point. Point out differences between your findings and others and then highlight what gap in literature was closed by your findings. Using literature, then explain why this is important. | We feel that the first two paragraphs of the discussion are important to include as presented. The first paragraph summarises the overall aims of the study and the second one summarises the main results, as is typically expected of a discussion section. The third paragraph includes the contributions of this study to the literature in the contexts of what is already known on the subject. |
| It is interesting that your findings suggest no concerns about negative health outcomes associated with vaping during pregnancy; however, the authors recommend “the development of alternative smoking cessation or reduction tools for particular use in pregnancy which limit harms to mother and baby.” First, what is a reduction tool? I wasn’t sure if the authors meant a harm reduction tool at first, but then thought this was referring to cessation methods. Next, if the authors found no associations with vaping during pregnancy and harm, why still suggest not using these devices? Unless the authors show the association between smoking during pregnancy and adverse health outcomes, it is unclear why the co-use of smoking and vaping could increase the prevalence of adverse pregnancy and birth health outcomes. | We have edited this to be clear that we mean “smoking-cessation or smoking-reduction tools”.  Our recommendation for alternative tools to aid smoking cessation and/or smoking reduction instead of recommending vaping to pregnant women, despite not finding an association with birth outcomes, is based on the following reasons which we have articulated in the introduction and discussion of the manuscript:   1. The totality of evidence on the known harms of exposure to nicotine (whether by vaping or otherwise) and emerging evidence of vaping associated lung damage in the general population would generally preclude a recommendation to vape. 2. This study observed more frequent cigarette smoking among women who vaped, and we cannot rule out the possibility that the more frequent smoking was a consequence of vaping; given (1) above, to recommend vaping would have the potential to increase potential harm to women and their infants. |
| **Minor comments:** Where it reads ‘raises a real risk…’ please remove ‘real’. | We have removed ‘real’ from this paragraph. |
| Some areas are wordy and confusing. For example, where it reads ‘and with that the emergence of reports of’, the authors could simply use ‘followed by reports of…’ | We have re-worded this sentence as suggested. |
| Commas appeared to be misused throughout the paper. For example, “…during pregnancy, and to women planning a pregnancy.” Also, the purpose statement does not need a comma. To use a common after the word “and”, there must be a noun and verb on both sides of the comma. The last sentence before analysis is missing punctuation. | We have now rechecked the punctuation throughout the manuscript to ensure their appropriate use. |
| What does ‘explained away’ mean? | It is a concept used to describe the nature of the association between a factor and an outcome conditional on a second factor.  If two factors are each associated with an outcome and to some extent with each other, then information about each factor’s association with the outcome is contained in the second factor. If a small amount of the information about a factor’s association with the outcome is contained in the second factor, then the first factor *explains* some of the second factor’s association with the outcome; this manifests in attenuation of the magnitude of the second factor’s association with the outcome when the first factor is adjusted for. If most or all of the information about a factor’s association with the outcome is contained in the second factor, then the second factor *explains away* the association between the first factor and the outcome, and this is seen in complete attenuation to the point of nullification of any association between the second factor and the outcome upon adjustment for the first factor.  For example, the pattern of smoking *explains away* the association between maternal characteristics and vaping because there is evidence of association between some maternal characteristics and vaping, but upon adjusting for the pattern of smoking this association is attenuated to the extent that it is nullified.  Nevertheless, we have replaced it with the alternative term ‘fully explains’ for readers not familiar with this term. |
| Avoid using terms such as ‘very’. | We have now edited the sentence where ‘very’ had been used to drop the word. |
| Again, some wordy sections included “It may also indicate women who were more frequent smokers were turning to vaping to aid smoking cessation but not being successful at quitting.” This could be rephrased to read “This may indicate participants who were more frequent smokers attempted to use vaping to quit smoking but were unsuccessful.” | We have adopted the suggested wording. |
